# Rapid Electron Transfer within the III-IV Supercomplex in *Corynebacterium glutamicum*

**DOI:** 10.1038/srep34098

**Published:** 2016-09-29

**Authors:** Simone Graf, Olga Fedotovskaya, Wei-Chun Kao, Carola Hunte, Pia Ädelroth, Michael Bott, Christoph von Ballmoos, Peter Brzezinski

**Affiliations:** 1 Department of Biochemistry and Biophysics, The Arrhenius Laboratories for Natural Sciences, Stockholm University, SE-106 91 Stockholm, Sweden; 2 Department of Chemistry and Biochemistry, University of Bern, Freiestrasse 3, 3012 Bern, Switzerland; 3 Institute of Biochemistry and Molecular Biology, ZBMZ, Faculty of Medicine, BIOSS Centre for Biological Signalling Studies, University of Freiburg, 79104 Freiburg, Germany; 4 IBG-1: Biotechnology, Institute of Bio- and Geosciences, Forschungszentrum Jülich, Wilhelm-Johnen-Strasse, D-52425 Jülich, Germany

## Abstract

Complex III in *C. glutamicum* has an unusual di-heme cyt. *c*_1_ and it co-purifies with complex IV in a supercomplex. Here, we investigated the kinetics of electron transfer within this supercomplex and in the cyt. *aa*_3_ alone (cyt. *bc*_1_ was removed genetically). In the reaction of the reduced cyt. *aa*_3_ with O_2_, we identified the same sequence of events as with other A-type oxidases. However, even though this reaction is associated with proton uptake, no pH dependence was observed in the kinetics. For the cyt. *bc*_1_-cyt. *aa*_3_ supercomplex, we observed that electrons from the *c*-hemes were transferred to Cu_A_ with time constants 0.1–1 ms. The *b*-hemes were oxidized with a time constant of 6.5 ms, indicating that this electron transfer is rate-limiting for the overall quinol oxidation/O_2_ reduction activity (~210 e^−^/s). Furthermore, electron transfer from externally added cyt. *c* to cyt. *aa*_3_ was significantly faster upon removal of cyt. *bc*_1_ from the supercomplex, suggesting that one of the *c*-hemes occupies a position near Cu_A_. In conclusion, isolation of the III-IV-supercomplex allowed us to investigate the kinetics of electron transfer from the *b*-hemes, via the di-heme cyt. *c*_1_ and heme *a* to the heme *a*_3_-Cu_B_ catalytic site of cyt. *aa*_3_.

The respiratory chain in aerobic organisms is composed of a number of membrane-bound protein complexes through which electrons, originating from the oxidation of organic compounds, are transferred to finally reach O_2_. The free energy released in this process is employed to establish a proton electrochemical gradient across the membrane, which is used to synthesize ATP by the F_1_F_O_ ATP synthase or for secondary transmembrane transport. In mitochondria, Complex III (the cytochrome (cyt.) *bc*_1_-complex) of the respiratory chain links the two-electron oxidation of quinol (QH_2_) to the one-electron reduction of water-soluble cyt. *c* in the respiratory chain:


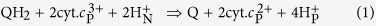


where the subscripts *N* and *P* refer to the more negative and positive sides of the membrane, respectively. Reduced cyt. *c* delivers electrons to Complex IV (cytochrome *c* oxidase, Cyt*c*O), which catalyzes the reduction of dioxygen to water:


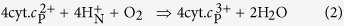


Part of the respiratory-chain enzymes in mitochondria are organized in so-called supercomplexes^1^,^2^,^3^,^4^,^5^,^6^,^7^,^8^,^9^,^10^,^11^. There are also reports of supercomplexes in bacteria, for example in *Paracoccus denitrificans*. In this bacterium, depending on the detergent used, supercomplexes composed of respiratory-enzyme complexes III-IV or I-III-IV at variable stoichiometries were identified^1^,^12^,^13^,^14^. Furthermore, in several bacterial systems electrons could be transferred directly between complexes III and IV via a membrane-anchored cyt. *c*^15^,^16^,^17^.

*Corynebacterium glutamicum* is a rod-shaped, Gram positive soil bacterium, which harbors two different terminal oxidases; an *aa*_3_*-*type Cyt*c*O and a *bd*-type menaquinol oxidase^18^,^19^,^20^. The cyt. *aa*_3_ in *C. glutamicum* is a four-subunit protein complex, comprising subunits CtaD, C, E, and F. Mass spectrometric analyses of the purified Cyt*c*O revealed that instead of heme *a*, the *C. glutamicum* contains heme *a*_s_ in the active site. Furthermore, the *C. glutamicum* Cyt*c*O harbors an extra charged amino-acid cluster near the cyt. *c*-binding domain of subunit II (CtaC), which was suggested to interact with the second cyt. *c* of the cyt. *bc*_1_ complex^18^.

Complex III in *C. glutamicum* is a three-subunit protein, containing cyt. *c*_1_ (QcrC), the Rieske iron-sulfur protein (QcrA), and cyt. *b* (QcrB) (Fig. 1A)^21^,^22^. The QcrC subunit contains two CXXCH heme-binding motifs, suggesting that this protein complex contains two *c*-type hemes^19^,^22^, hence referred to as the di-heme *c*_1_ cyt. *bc*_1_ complex. Furthermore, *C. glutamicum* contains no other *c*-type hemes, which suggested that the second heme *c* in cyt. *bc*_1_ shuttles electrons between complexes III and IV that form a tight supercomplex^21^. Such a supercomplex was isolated, its shape was determined using electron microscopy^23^ and it was shown to exhibit quinol-oxidase activity^20^:


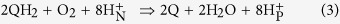


The involvement of the second cyt. *c* of cyt. *bc*_1_ in electron transfer between cyt. *c*_1_ and Cu_A_, the electron acceptor of CytcO (see below), is also supported by mutagenesis data^20^. Quinol-oxidase activity was also found for the cyt. *bc*_1_-*aa*_3_ supercomplex from *Mycobacterium smegmatis*^24^, a bacterium also devoid of soluble cyt. *c*.

The mechanism of the cyt. *bc*_1_ complex involves a Q-cycle in which the net reaction results in oxidation of menaquinol and reduction of cyt. *c* (see Fig. 1A), linked to proton uptake from the *N* side and release to the *P* side of the membrane. The process is initiated by binding of a menaquinol at the quinone-binding site, Q_P_, located near the low-potential heme *b*_L_ (see Fig. 1A)^25^. In the next step, one electron is transferred from the menaquinol, via the Rieske protein [2Fe–2S] cluster, to cyt. *c*_1_ while one electron is transferred via heme *b*_L_ and heme *b*_H_ to a menaquinone bound at a second quinone-binding site, Q_N_. This bifurcated electron transfer yields reduced cyt. *c*_1_, a semireduced menaquinone at the Q_N_-site and release of two protons to the *P*-side of the membrane. After binding a second menaquinol at the Q_P_-site the same process is repeated. The doubly reduced menaquinone at the Q_N_ site picks up two protons from the *N*-side of the membrane to form a menaquinol that is released into the membrane.

Cytochrome *c* oxidase, which belongs to a large family of enzymes called the heme-copper oxidases, catalyzes oxidation of cyt. *c* and reduction of O_2_ to H_2_O. Here, we refer to the *C. glutamicum* cytochrome *aa*_3_ as a Cyt*c*O even though in the cyt. *bc*_1_-cyt. *aa*_3_ supercomplex it receives electrons from quinol, via the cyt. *bc*_1_ complex and not from (a water-soluble) cyt. *c*. The heme-copper oxidases are classified according to sequence, phylogenetic, and structural analyses into three main classes, A, B and C^26^,^27^. Subunit I, the core subunit shared by all of the three oxidase types, contains a low-spin heme group and the catalytic site, which is composed of a copper ion, Cu_B_, and a high-spin heme (for review on structure and function of the Cyt*c*Os, see^28^,^29^,^30^,^31^,^32^,^33^,^34^,^35^,^36^,^37^). The fourth redox-active site, Cu_A_, is found in subunit II. The most studied Cyt*c*Os are those from bovine heart mitochondria and the bacterial *aa*_3_ Cyt*c*Os from *Paracoccus denitrificans* and *Rhodobacter sphaeroides*, which all belong to the A-class. In these Cyt*c*Os, electrons delivered by cyt. *c* are transferred consecutively to the Cu_A_ site, heme *a* and finally to the binuclear center composed of heme *a*_3_ and Cu_B_. In these bacterial Cyt*c*Os protons are transferred to the catalytic site through two pathways denoted by the letters D and K after conserved residues Asp132 and Lys362, respectively (numbering refers to the *R. sphaeroides aa*_3_-type Cyt*c*O).

Internal electron and proton-transfer reactions in Cyt*c*Os from several organisms have been studied in the past (see e.g.^30^,^31^,^34^,^37^). An experimental technique that yields particularly detailed information about the sequence and rates of these reactions is the so-called flow-flash technique, in which the oxidative part of a reaction cycle (single turnover) of the enzyme is monitored. In this approach, the oxidase is first fully reduced by four electrons (see dashed line in Fig. 2) and incubated under an atmosphere of carbon monoxide, which binds to heme *a*_3_ at the catalytic site. The Cyt*c*O-CO complex is then rapidly mixed with O_2_-containing solution followed in time by light-induced dissociation of the blocking CO ligand, which allows O_2_ from the surrounding medium to bind. Initially, the reduced Cyt*c*O (the state is called **R**) binds O_2_ to heme *a*_3_ with a time constant of ~10 μs (at 1 mM O_2_) yielding the ferrous heme *a*_3_-O_2_ state (state **A**) (see Fig. 2). Next, an electron is transferred from heme *a* to the catalytic site and the O=O bond is cleaved, resulting in formation of a ferryl intermediate that is called “peroxy” (**P**_**R**_) for historical reasons (τ ≅ 30 μs). A proton is then taken up to the catalytic site resulting in formation of the ferryl state, **F**, with a time constant of ~100 μs at pH 7. At the same time, an electron is transferred from Cu_A_ to heme *a* in a small fraction of the population (not shown in Fig. 2). Finally, the last electron is transferred from the Cu_A_-heme *a* equilibrium to the catalytic site forming the oxidized Cyt*c*O (state **O**) with a time constant of ~1 ms at pH 7 (the time constants are those observed with the *R. sphaeroides* Cyt*c*O^38^).

In the present study we used the flow-flash technique to investigate the reaction of the purified *C. glutamicum* Cyt*c*O, as well as the cyt. *bc*_1_-Cyt*c*O supercomplex with O_2_. The data indicate rapid electron transfer from the cyt. *bc*_1_-complex to the Cyt*c*O, suggesting a functional supercomplex in which the additional cyt. *c* of the cyt. *bc*_1_ complex acts as an electron bridge between the two respiratory-enzyme complexes. Furthermore, a comparison of the TMPD/cyt. *c*-driven O_2_-reduction activities with the cyt. *bc*_1_-Cyt*c*O complex and with Cyt*c*O alone, indicate that the second heme *c* of the cyt. *bc*_1_ complex does bind at the surface of the Cyt*c*O, presumably near Cu_A_. We also studied the electron-transfer kinetics in purified Cyt*c*O and found that reaction steps linked to proton uptake during O_2_ reduction displayed pH-independent kinetics, suggesting differences in the p*K*_a_ of residues involved in proton transfer, compared to other A-type Cyt*c*Os.

## Results

### Sequence Alignment and Homology Modeling

**Figure 1:**
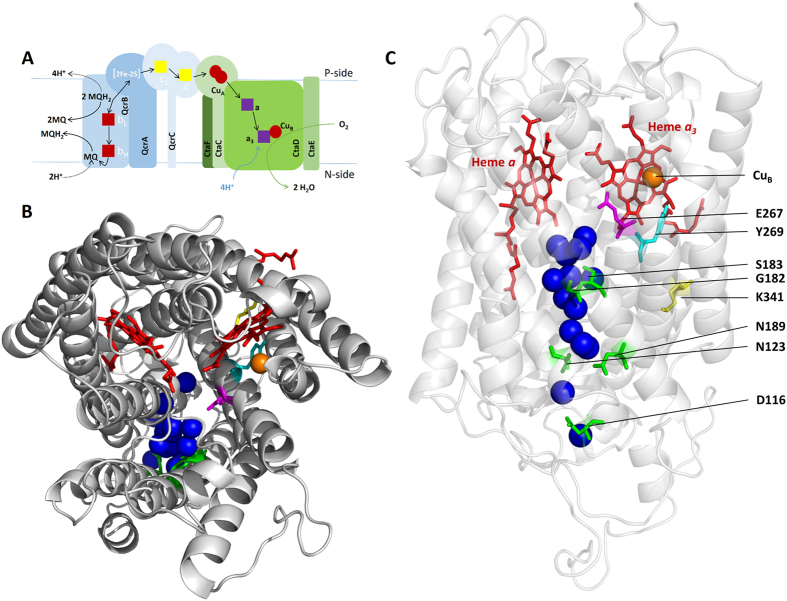
Schematic model of the *C. glutamicum* cyt. *bc*_1_-Cyt*c*O supercomplex and a homology model of subunit I in Cyt*c*O. (**A**) Cyt. *bc*_1_ (in blue) and Cyt*c*O (in green), represented as a supercomplex. The electron-transfer pathway is indicated by black bold arrows. Reduction of O_2_ at the heme *a*_3_-Cu_B_ catalytic site is accompanied by proton uptake from solution. The Figure is adapted from^19^. (**B,C**) A structural model of subunit I of CytcO from *C. glutamicum* (**B,C**, top and side view, respectively), based on the structure of *aa*_3_ (PDB ID 1M56) from *R. sphaeroides*^39^. Water molecules found in the structure of the *R. sphaeroides* Cyt*c*O are shown in the model to indicate the location of the D proton pathway (some residues defining this pathway are shown in green, Glu267 in magenta). Tyr269 is presumably part of the catalytic site. The putative K-pathway residue Lys341 (equivalent to Lys362 in *R. sphaeroides* Cyt*c*O) is shown as a yellow stick.

To analyze the structural characteristics of Cyt*c*O from *C. glutamicum,* we performed homology modeling of the highly conserved subunit I with the three-dimensional structure of that from the *R. sphaeroides aa*_3_-type Cyt*c*O^39^ using the SwissModel program^40^,^41^,^42^ (Fig. 1B,C). The two protein sequences are ~40% identical and ~60% similar. On the basis of the sequence itself and this model, the *C. glutamicum* protein is identified as an A1-type Cyt*c*O. It holds the active site tyrosine (Tyr269 in *C. glutamicum*)^21^ and Glu267 in the D pathway (Glu286 in *R. sphaeroides*). Furthermore, in the *C. glutamicum* Cyt*c*O an Asp residue (Asp116) is found at the same location as Asp132, the entry point of the D pathway in the *R. sphaeroides* Cyt*c*O. Other residues that are discussed below are Asn123 and Asn189 in the *C. glutamicum* Cyt*c*O (Asn139 and Asn207, respectively, in the *R. sphaeroides* Cyt*c*O).

### Purification and multiple turnover activity

We have purified the cyt. *bc*_1_-*aa*_3_ supercomplex from strain ΔC-D_St_ and the *aa*_3_ oxidase from the cyt. *bc*_1_ deficient strain ΔQ-D_St_. The quality of the preparations was assessed using SDS gel electrophoresis (Figure S1) and dithionite-reduced minus ferricyanide-oxidized difference spectra of the two samples (Figure S2). As expected, both spectra showed the heme *a* signature (605 nm), and the supercomplex preparation displayed additional peaks for *b*-heme (560 nm) and *c*-heme (550 nm) (Figure S2). The approximately similar height of the three alpha peaks indicates the presence of a 1:1 complex of cyt. *bc*_1_ (2 heme *b* and 2 heme *c*) and cytochrome *aa*_3_ (two heme *a*). The reduced CO-bound minus reduced difference spectrum (Figure S3) displayed the characteristic features of CO binding to heme *a*_3_.

Next, the quinol-oxidase activity of the purified supercomplex was measured by following O_2_ consumption after addition of a pre-reduced menaquinone. This process involves quinol oxidation by the cyt. *bc*_1_ complex followed by electron transfer to Cyt*c*O, where oxygen is reduced to water. The measured quinol-oxidase activity for the purified supercomplex was 210 ± 20 e^−^/s (SD, *n* = 4 measurements) (normalized to the total Cyt*c*O), i.e. in the same range as the published value^20^. The activity dropped rapidly upon flash freezing and thawing the preparation. Therefore, the sample was kept at 4 °C, where no activity loss was observed in the time frame between purification and functional studies (typically ~1 day, but the preparation was stable up to 7 days, Figure S4).

**Figure 3:**
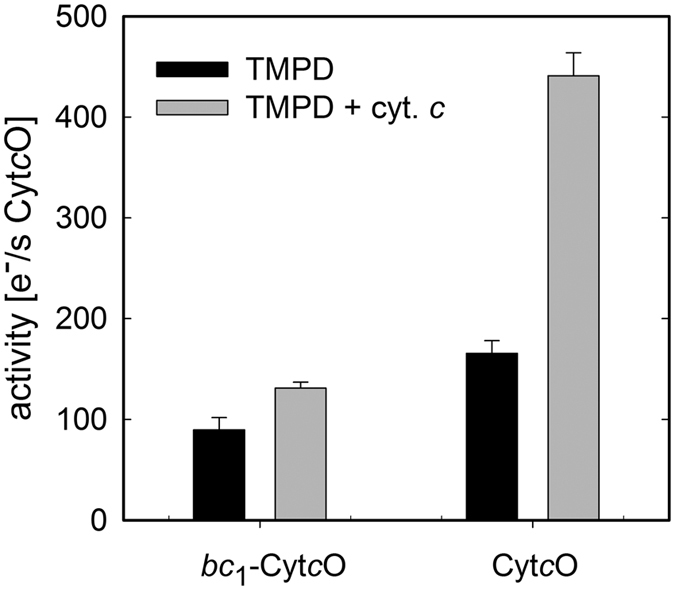
O_2_-reduction activity upon addition of an electron donor to Cyt*c*O. The measurements were done with either Cyt*c*O or the cyt. *bc*_1_-Cyt*c*O super complex using ascorbate as electron donor and, TMPD and cyt. *c* as electron mediators. The activity was determined by measuring the O_2_-reduction rate (e^−^/s/Cyt*c*O). Conditions: 100 mM Tris-HCl pH 7.5, 100 mM NaCl, 2 mM MgSO_4_, 0.015% (w/v) DDM, 2 mM sodium ascorbate, 0.2 mM TMPD, with or without 24 μM cyt.*c*.

Cyt*c*O activity of the purified supercomplexes and pure Cyt*c*O (without cyt. *bc*_1_) was also measured using ascorbate as electron source and either TMPD, or TMPD and water-soluble cyt. *c* as electron mediators (Fig. 3). For the cyt. *bc*_1_-Cyt*c*O, the TMPD activity was 90 ± 10 e^−^/s and it increased to 130 ± 10 e^−^/s (SD, *n* = 3) upon addition of free cyt. *c*, i.e. both rates were lower than the coupled quinol activity. For pure Cyt*c*O, the activity increased from 160 ± 10 e^−^/s to 440 ± 20 e^−^/s (SD, *n* = 3) upon addition of cyt. *c*, thus displaying a much greater stimulation by the soluble electron carrier.

### Kinetics of CO rebinding after flash photolysis

**Figure 4:**
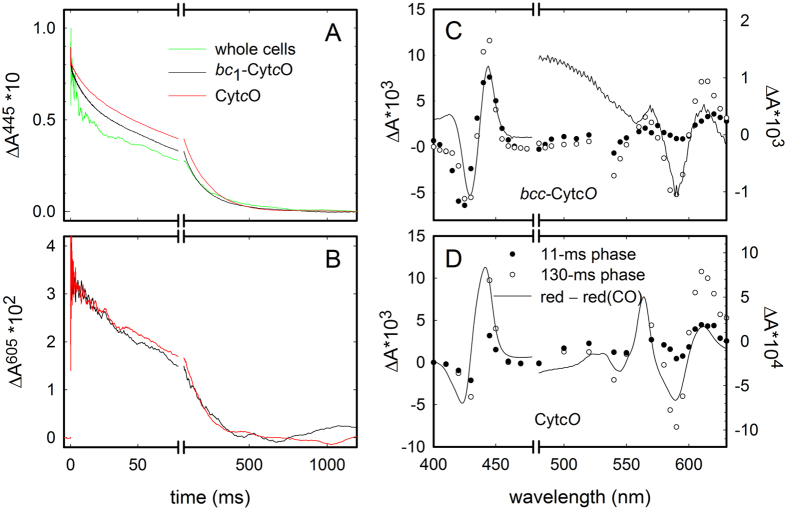
CO-recombination kinetics and kinetic difference spectra. Absorbance changes at 445 nm (**A**) and 605 nm (**B**) associated with light-induced CO dissociation and recombination with detergent-purified Cyt*c*O, cyt. *bc*_1_-Cyt*c*O and with the ΔC-D_St_ whole cells. All traces were scaled to yield the same absorbance changes at *t* = 0. A laser artifact at *t* = 0 has been removed for clarity. Kinetic difference spectra, i.e. the amplitude of the absorbance changes of the two kinetic components (slow and fast components, empty and filled circles, respectively), for Cyt*c*O (**C**) and the cyt. *bc*_1_-Cyt*c*O complex (**D**). Note the different ordinate scales before and after the break on the abscissa, respectively. The black line represents the reduced minus CO-bound difference spectrum. Experimental conditions: 100 mM Tris-HCl at pH 7.5, 1 μM PMS, 5 mM sodium ascorbate. For the cyt. *bc*_1_-Cyt*c*O supercomplex, 1 mM sodium dithionite was added to reduce the *b*-hemes.

CO binds with high affinity to heme *a*_3_ in the binuclear site in the reduced Cyt*c*O. Upon illumination with a short laser flash, CO dissociates instantly, but in the absence of oxygen rebinds to the binuclear site (CO recombination). The CO-recombination kinetics was biphasic where the relative contribution of the two components varied slightly between the purified Cyt*c*O, the cyt. *bc*_1_-Cyt*c*O complex and the intact membrane (Fig. 4A,B). This observation indicates the presence of two Cyt*c*O populations, also in the native membrane (see Discussion). The time constants for the fast and the slow phases were about the same for all samples (pure Cyt*c*O, the purified cyt. *bc*_1_-Cyt*c*O supercomplex and whole cells), i.e., 11 ± 3 ms and 130 ± 30 ms (SD, *n* = 15) (at 1 mM CO), respectively. The kinetic difference spectra of the fast and the slow components were similar (Fig. 4C,D).

The apparent binding affinity for CO to the binuclear center of the oxidase was determined by measuring the observed CO-recombination rates for the slower kinetic phase at different CO-concentrations, for both the oxidase alone and the purified supercomplex (Figure S5). The second-order rate constants, determined from a linear fit to the data in Figure S5, were 7.6 ± 0.2 *10^3^ M^−1^s^−1^ and 9.4 ± 0.2 *10^3^ M^−1^s^−1^ for the Cyt*c*O and cyt. *bc*_1_*-*Cyt*c*O complex, respectively. The rate of the faster component was CO-concentration independent.

### Single-turnover measurements

**Figure 5:**
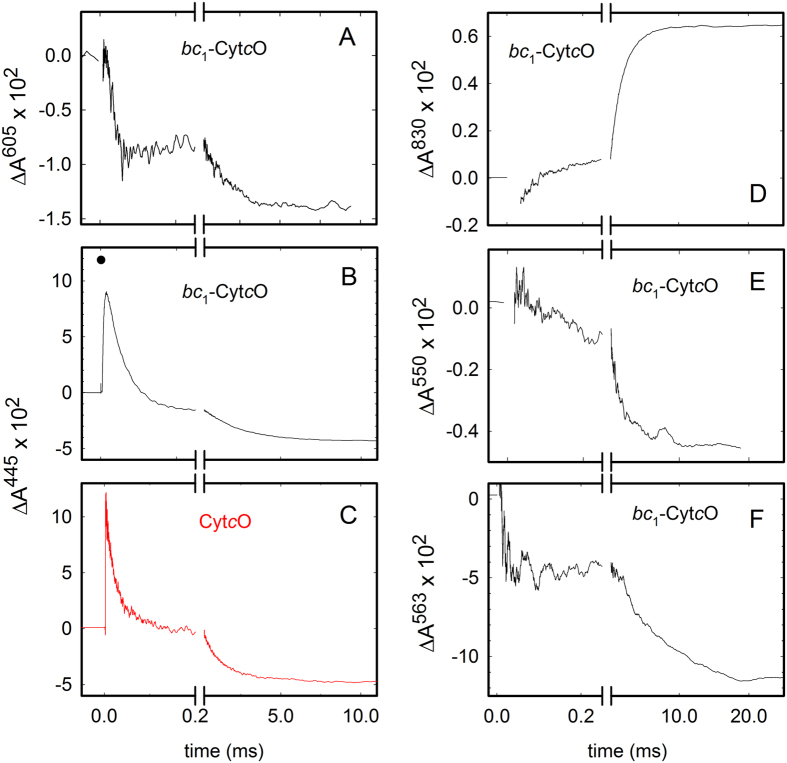
Absorbance changes associated with the reaction of the reduced cyt. *bc*_1_-Cyt*c*O complex and Cyt*c*O with O_2_. Absorbance changes were recorded over time at 605 nm (**A**), 445 nm (**B**) both associated with oxidation of *a*-hemes, and at 445 nm with the pure Cyt*c*O (**C**) (this trace was multiplied by a factor of two to yield approximately the same absorbance changes as those seen in (**B**). The absorbance changes at 830 nm (**D**) are associated with oxidation of Cu_A_, at 550 nm (**E**) oxidation of cyt. *c*, and at 563 nm (**F**) oxidation of the *b*-hemes. The CO ligand was dissociated at *t* = 0. Experimental conditions: 100 mM Tris-HCl at pH 7.5, 4 mM ascorbate, 1 μM PMS, ~900 μM O_2_. In addition, dithionite was added to fully reduce the cyt. *bc*_1_ complex. The Cyt*c*O concentration was ~1 μM. A laser artifact at *t* = 0 has been removed for clarity. The filled circle in (**B**) represents the extrapolated initial absorbance after the laser flash.

Figure 5 shows absorbance changes after flash-induced dissociation of the CO ligand from the reduced cyt. *bc*_1_-Cyt*c*O supercomplex in the presence of O_2_. At 605 nm (Fig. 5A), three kinetic phases were resolved. The initial decrease in absorbance, with a time constant of ∼25 μs [The standard deviation of the time constants was typically 10% of the measured values (*n* = 7–17), except for the **P** → **F** reaction for which the standard deviation was 20% of the measured values (for both the pure Cyt*c*O and the supercomplex)], is attributed to oxidation of heme *a*, i.e. electron transfer from heme *a* to heme *a*_3_, which yields the **P**_**R**_ state at the catalytic site (formation of state **A** was not resolved, see below). This component is also seen at 445 nm (initial decrease, Fig. 5B). At 605 nm, the decrease is followed by a small increase in absorbance in the time range 0.05–0.2 ms that is attributed to re-reduction of heme *a* with a time constant of 120 μs concomitant with the **P**_**R**_ → **F** reaction at the catalytic site. The slowest absorbance decrease (τ ≅ 1.7 ms) at 605 nm is associated with oxidation of the Cyt*c*O (**F** → **O)**, also seen at 445 nm (Fig. 5B). At 830 nm, oxidation of Cu_A_ is observed as an increase in absorbance. The small initial increase in absorbance is associated with oxidation of Cu_A_ during the **P**_**R**_ → **F** transition, i.e. τ ≅ 120 μs while the major oxidation component displayed a time constant of ~1.7 ms (Fig. 5D).

The absorbance changes at 550 nm (Fig. 5E) are mainly attributed to *c*-heme absorption, where a decrease in absorbance is associated with oxidation of the hemes. Two kinetic components with time constants of ~120 μs and ~1.7 ms, respectively, were observed, i.e. concomitant with electron transfer from Cu_A_ to heme *a* during the **P**_**R**_ → **F** reaction and during the **F** → **O** reaction. Finally, at 563 nm (Fig. 5F) after the unresolved initial drop in absorbance (presumably associated with a small absorbance contribution from CO dissociation), a further decrease in absorbance associated with oxidation of heme *b* (τ ≅ 6.5 ms) was observed, i.e. oxidation of heme *b* occurred after oxidation of the Cyt*c*O and the *c* heme (compare panels D-F in Fig. 5, after the break on the abscissa).

The concentration of reacting Cyt*c*O (i.e. Cyt*c*O from which the CO ligand is dissociated) was estimated from the change in absorbance at *t* = 0 at 445 nm (Fig. 5B), which yields ~0.15 μM Cyt*c*O (using an absorption coefficient (ε) of 82 mM^−1^cm^−1^ 43). The absorbance at *t* = 0^+^, i.e. just after CO dissociation corresponds to that of reduced Cyt*c*O. Consequently, the decrease in absorbance from this point until *t* ≅ 10 ms, when the reaction is essentially over, corresponds to the amount oxidized Cyt*c*O (~0.10 μM, using ε = 164 mM^−1^cm^−1 ^44). Consequently, ~0.05 μM (~35%) of the reacting Cyt*c*O becomes re-reduced by the cyt. *bc*_1_ complex. From the absorbance changes at 550 nm and 563 nm we estimate that ~0.025 μM (ε = 19.1 mM^−1^cm^−1^ 20) heme *b* and ~0.028 μM (ε = 22 mM^−1^cm^−1^ 20) heme *c*, respectively, become oxidized, which together account for ~0.05 μM Cyt*c*O that is re-reduced during the experiment. It should be noted that these estimations are only approximate because we were not able to accurately resolve O_2_ binding to the reduced heme *a*_3_ in all samples. In part, this problem is attributed to the requirement to add dithionite in order to fully reduce the cyt. *bc*_1_-Cyt*c*O supercomplex. Because dithionite reduces O_2_ directly during mixing, the O_2_ concentration was lowered before initiation of the reaction of Cyt*c*O with O_2_ thereby slowing the **R** → **A** reaction. Consequently, it was difficult to resolve the associated absorbance changes from those associated with the next, **A** → **P**_**R**_ transition.

The single-turnover reaction was also studied with purified Cyt*c*O (Fig. 5C). Here, heme *a* was oxidized with a time constant of ~21 μs (**R** → **P**_**R**_), followed in time by re-reduction from Cu_A_ with a time constant of ~90 μs (**P**_**R**_ → **F**) and oxidation with a time constant of ~1.3 ms (**F** → **O**). These time constants were almost the same as those observed with the cyt. *bc*_1_-Cyt*c*O supercomplex. No changes in absorbance were observed at 550 nm nor 563 nm for the purified oxidase (data not shown). The end absorbance level at 445 nm was slightly lower with pure Cyt*c*O than with Cyt*c*O that is part of the supercomplex (c.f. Fig. 5B,C), which means that the former was more oxidized than the latter. This observation presumably reflects the re-reduction of Cyt*c*O by the cyt. *bc*_1_ complex in the latter. However, as mentioned above, it was difficult to quantify the relative absorbance differences in the two samples because we were unable to confidently scale the two different traces to each other due to unresolved absorbance changes associated with O_2_ binding.

Binding of water-soluble cyt. *c* to e.g. the bovine heart Cyt*c*Os is highly dependent on the salt concentration, reflecting electrostatic interactions of the two proteins^45^. In order to investigate whether or not the interactions of Cyt*c*O with cyt. *c* in the supercomplex could be disrupted, we studied the reaction with O_2_ at increasing ionic strengths (addition of KCl). As seen in Figure S6, the amplitude of the absorbance changes associated with the **F** → **O** reaction decreased slightly (indicating more re-reduction of CytcO) rather than increasing with increased ionic strength (we expect more oxidation of Cyt*c*O upon cyt. *c* dissociation), which indicates that the cyt. *c*-Cyt*c*O interactions were not disrupted at high salt concentrations.

### Net Proton Uptake from Solution

**Figure 6:**
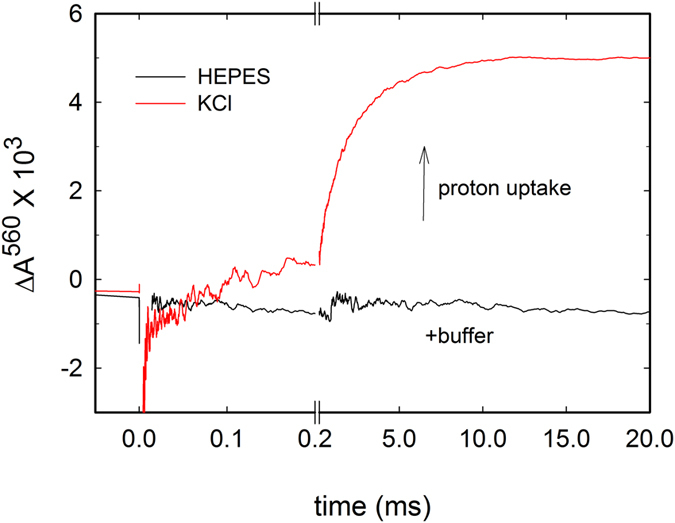
Absorbance changes of the pH dye phenol red, associated with proton uptake during O_2_ reduction. The reaction of the reduced cyt. *bc*_1_-Cyt*c*O complex with O_2_ was initiated by a laser flash at *t* = 0 (see Fig. 5). Absorbance changes were monitored over time at 560 nm in the absence and presence of buffer, respectively. Experimental conditions: 0.05% DDM, 100 μM EDTA, 40 μM Phenol red at pH 7.8 2 mM ascorbate, 0.2 μM Hexamminerutheniumchloride, ~900 μM O_2_ and either 100 mM KCl or 100 mM HEPES. The Cyt*c*O concentration was ~5 μM. A laser artifact at *t* = 0 has been removed for clarity.

The protons required for the reduction of O_2_ to water are taken up from the medium, a process that can be monitored during flow-flash experiments in the absence of buffer by use of a pH-sensitive dye. Using phenol red, we studied proton uptake during reaction of the reduced cyt. *bc*_1_-Cyt*c*O supercomplex with O_2,_ following absorbance changes at 560 nm. As seen in Fig. 6, the absorbance increased over time, which indicates net proton uptake during the reaction. The process displayed two components with time constants of 130 μs (~25% of the total absorbance change) and 1.9 ms, i.e. they coincided with the **P**_**R**_ → **F** and **F** → **O** reactions, respectively. Upon addition of buffer the dye signal was quenched and only a very small decrease in absorbance associated with oxidation of *b*-hemes was observed (c.f. Fig. 6).

### pH-dependence

**Figure 7:**
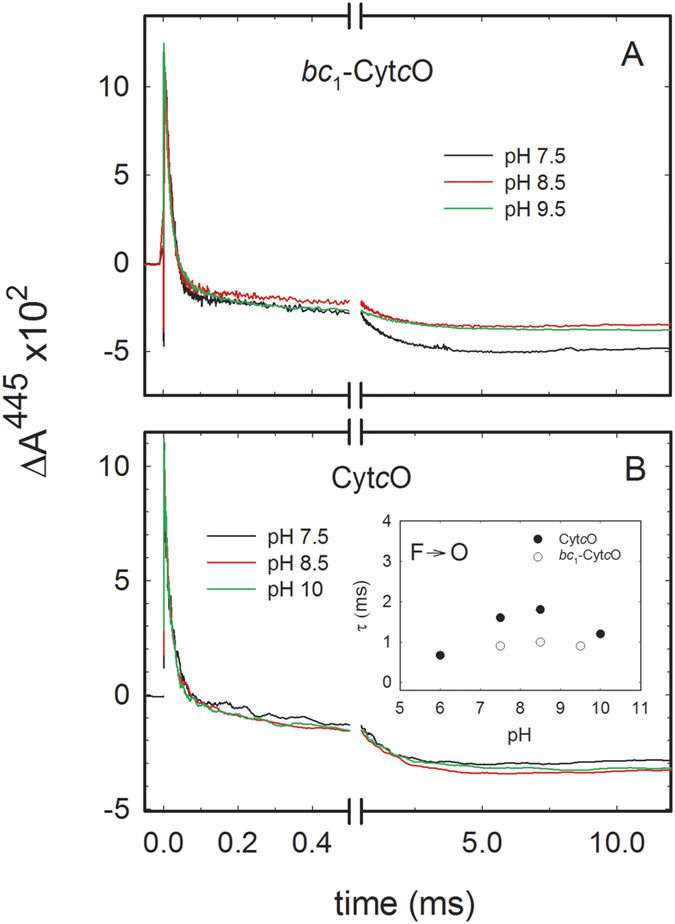
pH dependence of the reaction of cyt. *bc*_1_-Cyt*c*O (**A**) and Cyt*c*O (**B**) with O_2_. Kinetic data at representative pH-values are shown (color codes as indicated in the graph) and the time constants are summarized in the inset to panel B (measurements were also performed at pH 6 with the Cyt*c*O). The CO ligand was dissociated at *t* = 0. Experimental conditions: ~1 μM Cyt*c*O, 10–100 mM Tris-HCl (see Materials and Methods), 4 mM ascorbate, 1 μM PMS and 100 μM dithionite. The pH was set as described in the Materials and Methods section, and measured after mixing using a pH meter. A laser artifact at *t* = 0 has been removed for clarity.

Reactions steps that are associated with proton uptake from solution (e.g. the **F** → **O** step of the reaction of reduced CytcO with O_2_) often display pH dependent rates^30^,^46^,^47^. We therefore investigated the pH dependence of the reaction with O_2_ of the reduced cyt. *bc*_1_-Cyt*c*O supercomplex and Cyt*c*O alone (Fig. 7A,B). At 445 nm, for the supercomplex, only a slight pH dependence was observed for the **F** → **O** reaction rate. As the total amplitude of the oxidation also changed, the results presumably reflect a pH-dependence in the extent of re-reduction by the cyt. *bc*_1_ complex, i.e. “down-stream” steps of the reaction. Also with the pure Cyt*c*O, the **F** → **O** reaction rate was pH independent and all kinetic components displayed essentially the same amplitudes in the measured pH range (c.f. data at pH 7.5 and 8.5 in Fig. 7B).

### Na^+^ -dependence

Because we did not observe any significant pH-dependence in the reaction rates with O_2_, we also investigated the Na^+^ -concentration dependence to test the possibility that the Cyt*c*O transports Na^+^ (see refs 48 and 49). As can be seen in Figure S7, the addition of Na^+^ had a slight effect on the time constant of the **A** → **P**_**R**_ reaction, i.e. electron transfer from heme *a* to the catalytic site (inset Figure S7), but this reaction step is not linked to pumping in other A-type oxidases. No effect on the kinetics of the **F** → **O** reaction was observed.

## Discussion

### Purification and Activity

The purified cyt. *bc*_1_-Cyt*c*O complex displayed quinol-oxidase activity, i.e. electrons were transferred first from quinol to the cyt. *bc*_1_ complex and then, via the two *c*-hemes^20^, to the Cyt*c*O, which reduces oxygen to water. The activity was ~210 e^−^/s, which is in good agreement with previously published results^20^. The data are also consistent with those obtained for the cyt. *bc*_1_-Cyt*c*O supercomplex from *Mycobacterium smegmatis*^24^, which exhibited quinol-oxidase activity even at high detergent concentrations, supporting the presence of the cyt. *bc*_1_-Cyt*c*O supercomplex. Both the *C. glutamicum* cyt. *bc*_1_-Cyt*c*O and Cyt*c*O preparations exhibited TMPD-driven O_2_-reduction activities. In both cases the activity increased upon addition of soluble cyt. *c*. However, the increase was larger for the pure Cyt*c*O (a factor of ~2.8) than for the supercomplex (a factor of ~1.4) (but see data with the *M. smegmatis*^24^). This observation suggests that in *C. glutamicum* cyt. *c* is more accessible for binding to the Cyt*c*O upon removal of the cyt. *bc*_1_ complex, which indicates that in the supercomplex one of the *c*-hemes is located near the Cyt*c*O electron entry point (*i.e.* presumably near Cu_A_). Thus, the results indicate that pure Cyt*c*O (i.e. with the cyt. *bc*_1_ removed) is capable of binding soluble cyt. *c* in spite of the presence of an extra loop of charged amino acids located at the cyt. *c* binding site^18^ (this binding can only occur in the mutant where the cyt. *bc*_1_ complex is removed, i.e. not *in vivo*). Formation of a stable cyt. *bc*_1_-Cyt*c*O complex that is capable of transferring electrons directly from cyt. *bc*_1_ to Cyt*c*O is also consistent with the rapid electron transfer from the *b* and *c*-hemes to Cyt*c*O (see below).

We note that the TMPD and cyt. *c*-oxidation activities measured here are higher than those presented previously^18^,^20^. The discrepancy is presumably due to differences in experimental conditions. While in the earlier studies the rate was derived from changes in the concentration of reduced electron donor, here the data was obtained by measuring the O_2_-reduction rate at a constant concentration of the reduced electron donor (with excess ascorbate).

### Ligand Binding to Cyt*c*O

Results from earlier studies with e.g. the bovine heart oxidase indicate that upon pulsed illumination the CO ligand dissociates from heme *a*_3_ and binds transiently to Cu_B_ before it dissociates into solution^50^:


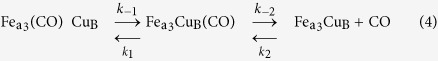


Dissociation of CO from heme *a*_3_ in the dark is very slow (*k*_−1_ ≅ 0.03 s^−1^), but upon illumination the ligand moves to Cu_B_ in << 10 ns, if the light intensity of the pulse is strong enough (such as in this study). The dissociation rate constant from Cu_B_, k_−2_, is ~7·10^5 ^s^−1^ with the bovine heart Cyt*c*O^50^. Recombination of CO occurs via Cu_B_ with a second-order process (*k*_2_ ≅ 1·10^8^ M^−1^s^−1^). The rate for internal CO transfer from Cu_B_ to heme *a*_3_, *k*_1_, is ~10^3 ^s^−1^. The observed rate of CO recombination is approximately given by the fraction of Cu_B_ with bound CO (middle state in scheme 1) multiplied by the rate of CO transfer from Cu_B_ to heme *a*_3_,


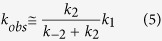


With the rate constants given above, we obtain *k*_obs_ ≅ 0.12 × 1000 s^−1^ = 120 s^−1^ (τ ≅ 8 ms) at 1 mM CO.

The CO-recombination kinetics measured with the *C. glutamicum* Cyt*c*O was biphasic (see Fig. 4A). The kinetic difference spectra of the two components with the purified supercomplex and the oxidase alone were similar (see Fig. 4C,D), indicating that both components are associated with CO binding to heme *a*_3_ after light-induced dissociation. Consequently, the data indicate the presence of two populations of Cyt*c*O with different CO-recombination rates to heme *a*_3_. The smaller (~25%) Cyt*c*O population, displayed a CO-concentration independent rate constant of ~90 s^−1^ (also the relative amplitude was independent on the CO concentration). Assuming the model in Eq. 4, this observation indicates that for this population the ratio *k*_2_/(*k*_2_ + *k*_−2_) is equal to ~1 at all CO concentrations used in this study and that *k*_1_ = 90 s^−1^. Alternatively, after photolysis from heme *a*_3_ and binding to Cu_B_, the CO ligand did not equilibrate with solution in this population.

The slower, major CO-recombination component displayed a CO-concentration dependent rate constant of 6.7 s^−1^ at 1 mM CO. Assuming that *k*_1_ has the same value of 90 s^−1^ for the two Cyt*c*O populations, the ratio *k*_2_/(*k*_2_ + *k*_−2_) ≅ 0.07. Thus, the difference between the two Cyt*c*O populations reflecting the two time constants could be explained by different values of *k*_2_/(*k*_2_ + *k*_−2_), i.e. by differences in CO binding to Cu_B_. Because CO-recombination was biphasic both in whole cells and in the detergent-purified samples, the presence of the two components is not an artifact caused by the purification of the supercomplex or the oxidase. Instead, we speculate that the two components reflect two Cyt*c*O populations that are present in the native membrane and that could differ, for example, in the local structure of Cu_B_ resulting in different CO-binding affinities. Most likely these two Cyt*c*O populations would display different reactivity towards the natural ligand and electron acceptor, O_2_. If the relative fraction of these two populations would be modulated by the cell, this mechanism could be used to regulate electron transfer through the respiratory chain.

### Reaction with O_2_

The four-electron reduction of O_2_ to H_2_O takes place in a number of distinct kinetic steps in which the Cyt*c*O is gradually oxidized and O_2_ is reduced. We identified the kinetic components on the basis of a comparison to data obtained earlier with other well-studied oxidases^38^. With the pure Cyt*c*O we observed electron transfer from heme *a* to the catalytic site and formation of the “peroxy” state, **P**_**R**_, with a time constant of ~21 μs. Formation of the next, ferryl intermediate (**F**) displayed a time constant of 90 μs. Finally, the Cyt*c*O was oxidized forming the oxidized state (**O**) with a time constant of 1.3 ms. All these time constants are essentially the same as those observed previously with e.g. the well-studied Cyt*c*O from bovine heart or *R. sphaeroides*. Consequently, the differences in CO-binding kinetics between the earlier studied A-type oxidases and *C. glutamicum* Cyt*c*O are apparently not reflected in the kinetics of O_2_ binding and reduction.

**Figure 8:**
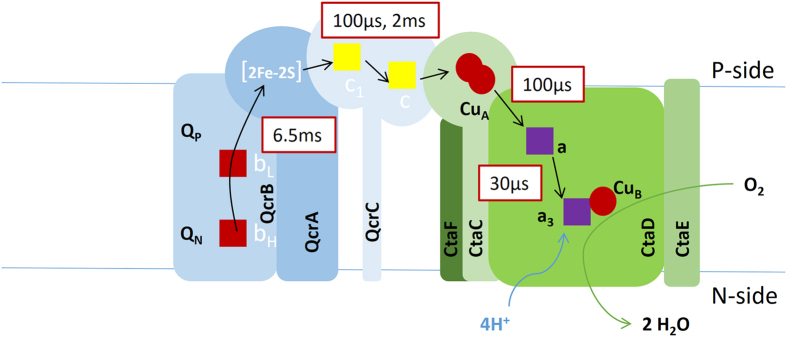
A schematic picture showing the approximate time constants for the electron-transfer reactions. Note that the positions of the heme groups in this picture are not compatible with the X-ray structure (e.g.^39^); they have been adjusted for clarity. Because the data cannot discriminate between the two heme *c*s or the two heme *b*s, we have indicated the oxidation time constants for each type of heme without specifying which heme that is oxidized. Redox reactions of the quinones are not observed in these experiments.

For the cyt. *bc*_1_-Cyt*c*O complex, all time constants for the different reaction steps were similar to those observed with the pure Cyt*c*O, however, there were also notable differences reflecting intra-complex electron transfer. During the **P**_**R**_ → **F** reaction (τ ≅ 120 μs) an electron is transferred from Cu_A_ to heme *a*, which leaves Cu_A_ oxidized allowing electron transfer from cyt. *c* of the cyt. *bc*_1_ complex to Cu_A_. This electron transfer is rate-limited by proton uptake^51^ and does not occur at the same rate as when photochemically injected into Cu_A_^52^. As seen at 550 nm (Fig. 5E), cyt. *c* was partially oxidized over a time scale of ~100 μs, which indicates that Cu_A_ was re-reduced concomitantly with the Cu_A_-to-heme *a* electron transfer. This interpretation is also supported by the very small extent of net Cu_A_ oxidation (observed at 830 nm) over the 100-μs time scale (Fig. 5D, see the small increase in absorbance). In the next step of the reaction, **F** → **O** (τ ≅ 1.7 ms), the fourth electron is transferred to the catalytic site, which allows further electron transfer from heme *c* to the Cyt*c*O, reflected in a further decrease in absorbance at 550 nm over the time scale of the **F** → **O** reaction in Cyt*c*O. The absorbance changes at 550 nm reflect oxidation of cyt. *c*, but we could not determine the degree of oxidation of each of the two cyt. *c*s separately. Most likely these two cyt. *c*s are oxidized simultaneously, but not necessarily to the same degree. In the summarizing Fig. 8 we indicate that both cyt. *c*s are oxidized over time scales of 100 μs and 1.7 ms (approximated by 2 ms in the figure).

We also observed further electron transfer from heme *b* (absorbance decrease at 563 nm, Fig. 5F), which reflects electron transfer from heme *b* to the Cyt*c*O, but this electron transfer significantly lags behind (τ ≅ 6.5 ms) that of the **F** → **O** reaction (τ ≅ 1.7 ms). As described above for cyt. *c*, also for heme *b* oxidation we could not discriminate between hemes *b*_L_ or *b*_H_ and conclude only that heme *b* of the cyt. *bc*_1_ is oxidized over the 6.5-ms time scale. As outlined in the Results section, the amounts of oxidized heme *c* and heme *b* approximately equal the amount of Cyt*c*O that becomes re-reduced during or after reaction with O_2_. Furthermore, the electron-transfer time constant from heme *b* to Cyt*c*O (τ ≅ 6.5 ms) is approximately compatible with the overall quinol-oxidation/O_2_ reduction turnover rate of the cyt. *bc*_1_-Cyt*c*O supercomplex (~210 s^−1^). This rapid electron transfer with a time constant of ~6.5 ms corresponds to a maximum electron-transfer rate over a distance of ~25 Å^53^. Even though the distances connecting the heme *c*s with their partners are not known for the *C. glutamicum* cyt. *bc*_1_-Cyt*c*O complex, this distance estimation is consistent with that between heme *b*_L_ and the iron-sulfur cluster in cyt. *bc*_1_ from e.g. *S. cerevisiae*^25^.

To investigate the functional stability of the cyt. *bc*_1_-Cyt*c*O supercomplex we investigated the reaction with O_2_ upon increasing the ionic strength (Figure S6). Neither the amplitudes nor rates of the observed absorbance changes at 445 nm were significantly altered even at the highest KCl concentration of 1.5 M. These results suggest that the purified supercomplex is stable and remains functionally intact. Furthermore, the interactions between one of the *c* hemes of the cyt. *bc*_1_ complex and Cyt*c*O seem more stable than those observed for the water-soluble cyt. *c* and Cyt*c*O from e.g. bovine heart as in the latter case the 1:1 cyt. *c*-Cyt*c*O complex dissociates at ionic strength above ~300 mM^45^.

### pH (in)dependence of the Reaction with O_2_

As seen in Fig. 7 we did not observe any significant pH-dependence in the kinetics of the **F** → **O** reaction, neither for the pure Cyt*c*O nor for the cyt. *bc*_1_-Cyt*c*O supercomplex. Slight differences were observed for the two samples (see inset to Fig. 7), which may be attributed to binding of cyt. *bc*_1_ to Cyt*c*O in the supercomplex. The observation that the **F** → **O** rate is essentially pH-independent is surprising given that the reaction is associated with proton uptake (see Fig. 6) and therefore expected to display pH-dependent kinetics, as in other oxidases^30^,^46^,^47^. One possible explanation for pH-independent kinetics of a reaction step that is linked to proton uptake is that the p*K*_a_ in this pH dependence may be outside of the accessible pH range. Alternatively, the proton uptake may not be part of the rate-limiting step, however, this explanation is less likely based on results from earlier studies with other A- and B-type oxidases where proton uptake is rate limiting^51^,^54^,^55^,^56^,^57^.

In earlier studies with the *R. sphaeroides* Cyt*c*O it has been observed that the **P**_**R**_ → **F** rate is essentially pH-independent up to pH ~9 and then decreases with increasing pH with an apparent p*K*_a_ of 9.4^58^. The **F** → **O** rate in *R. sphaeroides* Cyt*c*O displayed a more complex pH dependence and was found to titrate with two p*K*_a_s of ~9 and <6, respectively^30^. The p*K*_a_ around 9 was attributed to residue Glu286 within the D proton pathway, which is conserved in the *C. glutamicum* Cyt*c*O (Glu267). In the *R. sphaeroides* Cyt*c*O, replacement of e.g. Asn139 or Asn207 by Asp resulted in an increase in the Glu286 p*K*_a_; for example in the Asn139Asp variant the p*K*_a_ increased to a value above the accessible pH range^46^,^59^,^60^. In other words, even though the reaction is associated with proton uptake in these structural variants, it did not display a pH-dependent kinetics. In the *C. glutamicum* Cyt*c*O, many but not all residues “below” the Glu267 in the D pathway are conserved. The data with the *R. sphaeroides* structural variants show that very small changes in the D pathway structure, also at a distance from the Glu, may yield pH-independent kinetics. Thus, considering the differences in the environment of Glu267 in the *C. glutamicum* Cyt*c*O it is possible that it’s p*K*_a_ is tuned to adopt a value that is higher than that of Glu286 in the *R. sphaeroides* Cyt*c*O. However, more experiments with single point mutations in the D pathway are necessary to confirm this speculation.

## Concluding Remarks

The reaction of the purified reduced Cyt*c*O with O_2_ displayed the same sequence of electron transfers as that observed previously with other bacterial and mitochondrial A-type Cyt*c*Os. However, in contrast to data obtained with the other oxidases, none of the reaction steps associated with proton uptake displayed any pH dependent rates, which is explained in terms of an elevated apparent p*K*_a_ of Glu267. The data indicate that one of the *c* hemes of cyt. *bc*_1_ is bound near Cu_A_, at the same site where externally added water-soluble cyt. *c* would bind. Furthermore, the data indicate that the interaction of this heme *c* with Cyt*c*O is strong in the cyt. *bc*_1_-Cyt*c*O supercomplex and insensitive to changes in ionic strength. The second heme *c* of the cyt. *bc*_1_-Cyt*c*O supercomplex provides a link for direct electron transfer between cyt. *bc*_1_ and Cu_A_, the electron acceptor of Cyt*c*O (see Fig. 8), which was also indicated from earlier mutagenesis data^20^. Electron transfer from heme *c* to the Cyt*c*O occurred over time scales of approximately 100 μs–2 ms, while re-reduction of heme *c* by heme *b* displayed a time constant of ~6.5 ms, which suggest that this reaction may be rate limiting for the overall quinol-oxidation O_2_-reduction turnover rate. In conclusion, the isolation of a stable supercomplex from *C. glutamicum* allowed us to investigate the kinetics of electron transfer all the way from heme *b* in the cyt. *bc*_1_ complex to the catalytic site of Cyt*c*O, via the bridging *c* hemes.

## Materials and Methods

If not stated otherwise, the chemicals were purchased from Sigma-Aldrich.

### *Corynebacterium glutamicum* strains

The *C. glutamicum* strains used for purification of CytcO and the cyt. *bc*_1_-Cyt*c*O supercomplex were described before^20^. The ΔC-D_St_ strain refers to the 13032Δ*ctaD* strain transformed with the pJC1-*ctaD*_St_ plasmid (Kan^R^), which serves as an expression plasmid for Strep-tagged CtaD; *ctaD* is expressed from its native promoter and contains 10 additional codons at the 3′-end (AAWSHPQFEK)). The ΔQ-D_St_ strain refers to the 13032Δ*qcrCAB* strain transformed with the pJC1-*ctaD*_St_ plasmid.

### Culture Conditions

The cells were cultivated at 30 °C in all steps. Single *C. glutamicum* colonies were picked from BHI-Agar plates (33 g/l brain heart infusion broth, 15 g/l agar agar, 20 g/l D-(+)-glucose, 25 mg/l kanamycin) and inoculated into 10 ml BHI culture medium (33 g/l brain heart infusion broth, 20 g/l D-(+)-glucose, 25 mg/l kanamycin) at 220 rpm. After overnight growth the pre-culture was inoculated into 500 ml CGXII medium^20^ in a 2 l Erlenmeyer flask (at 160 rpm). After the optical density at 600 nm (OD_600_) reached between 25 and 30, the cells were diluted 1:20 into 2 l of CGXII medium into a 5 l baffled Erlenmeyer flask (at 130   rpm) and cultivated to an OD_600_ of 15–17 before harvest.

### Membrane Preparation

The cells were harvested using a Beckman centrifuge equipped with the JLA 8.1000 rotor at 7,500 rpm (~10,000 × g) for 30 min. The cells were homogenized in 4 ml cell lysis buffer (100 mM Tris-HCl pH 7.5, 5 mM MgSO_4_, some crystals phenylmethanesulfonyl fluoride, some crystals DNaseI (Roche)) per 1 g of cells (wet weight), and passed through a cell disrupter 4 times at 40 kPsi (Constant Systems). Cell debris was collected by centrifugation at 27,000 × g for 20 min. (Type 45Ti rotor, Beckman) and subsequently the membranes were collected by ultracentrifugation at 150,000 × g for 90 min (Type 45Ti rotor, Beckman).

### Protein Purification

The purification was done essentially as described in ref. 20 with minor modifications. Briefly, the membranes were mixed with solubilization buffer (100 mM Tris-HCl pH 7.5, 100 mM NaCl, 2 mM MgSO_4_, 50 mg/l avidin (iba lifescience), 1% (w/v) DDM (GLYCON Biochemicals) at a protein concentration of 5 mg/ml, and incubated at 4 °C for 45 min under slow stirring. Unsolubilized material was removed by ultracentrifugation (180,000 × g, 20 min, 4 °C). The supernatant was collected and concentrated in an Amicon Ultra 15 ml filter spin tube with 100 kDa cutoff until the volume was around 5 ml. The concentrated supernatant was diluted tenfold in solubilization buffer without DDM (yielding a final DDM concentration of 0.1% (w/v) and concentrated again to reach a volume <15 ml. The concentrated supernatant was applied to a Gravity flow Strep-Tactin Superflow column (bed volume 5 ml, iba lifescience). Subsequently, the column was washed 3 times with 0.5 column volumes of washing buffer (100 mM Tris-HCl pH7.5, 100 mM NaCl, 2 mM MgSO_4_, 0.015% (w/v) DDM) and protein was eluted with up to 3 column volumes elution buffer (100 mM Tris-HCl pH 7.5, 100 mM NaCl, 2 mM MgSO_4_, 0.015% (w/v) DDM, 2.5 mM D-desthiobiotin) and concentrated as described above. The protein samples (Cyt*c*O alone green colored, *bc*_1_-Cyt*c*O brown colored) were stored at 4 °C.

The Cyt*c*O alone, as well as the cyt. *bc*_1_*-*Cyt*c*O supercomplex, could be purified from membranes originating from the ΔQ-D_St_ and the ΔC-D_St_ *C. glutamicum* strains, respectively, both having a Strep-tag on subunit I of the Cyt*c*O. The SDS-PAGE for the two purifications (supplementary Figure S1) shows that the eluate of the supercomplex purification contains the three core subunits of Cyt*c*O (CtaD, C, and E), along with the three subunits of the *bc*_1_-complex (QcrC, A, and B), as well as some additional subunits, which were also co-purified with the supercomplex^20^. Accordingly, the eluate for the oxidase purification from ΔQ-D_St_ membranes contained only the three core subunits of the Cyt*c*O.

Dithionite-reduced minus ferricyanide-oxidized difference spectra were recorded at room temperature using a Cary100 UV-Vis Spectrophotometer. The Cyt*c*O concentration was determined from the reduced minus oxidized difference spectrum using the absorption coefficient Δε^600–630^ = 3.2 mM^−1^cm^−1^ 20.

### Quinone Reduction

Instead of ubiquinone, *C. glutamicum*, as a Gram-positive bacterium, employs menaquinones as an electron carrier in the membrane^61^. To prepare reduced quinol, 3.7 mg 2,3-dimethyl-[1,4]naphthoquinone (Rare Chemicals GmbH) was dissolved in 1 ml N_2_-saturated anhydrous cyclohexane to yield a 20 mM solution. The solution was mixed with 5 ml N_2_-saturated 1 M sodium dithionite solution (in H_2_O) and shaken vigorously. After phase separation, the organic phase containing the reduced 2,3-dimethyl-[1,4]naphthoquinol was removed and transferred to a 15 ml Falcon tube (all steps performed under a stream of N_2_). The cyclohexane was evaporated under an N_2_ stream, while the sample was kept at ~40 °C in a water bath. Subsequently, the reduced quinol was dissolved in N_2_-saturated, acidified ethanol (ethanol with 10 mM HCl), aliquoted, and flash frozen in liquid nitrogen, and stored at −20 °C.

### Determination of enzyme activities

Oxygen consumption in multiple turnover experiments was measured using a Clark-type oxygraph (Hansatech). The TMPD (N,N,N′,N′-tetramethyl-p-phenylenediamine)-oxidase activity was measured in 100 mM Tris-HCl (pH 7.5), 100 mM NaCl, 0.015% (w/v) DDM, 0.2 mM TMPD, 2 mM sodium ascorbate. Both TMPD and ascorbate were added before the sample and background oxygen consumption was measured and the reaction was started by adding the protein sample. Cytochrome *c* driven oxidase activity was measured as above in the presence of ascorbate and TMPD, but bovine heart cyt. *c* (25 μM final concentration) was also supplied before addition of the sample. Quinol-driven oxidase activity was measured in the same buffer (100 mM Tris-HCl (pH 7.5), 100 mM NaCl, 0.015% (w/v) DDM), but 20 μl of the reduced quinol solution (see above) was added before the protein sample. Background oxygen consumption was measured, followed by addition of the protein sample and monitoring the enzymatic oxygen consumption. When measuring the Cyt*c*O activity with ascorbate/TMPD/cyt. *c* as a substrate, this activity was insignificant. However, in the presence of quinol the background O_2_-reduction rate increased significantly, due to quinol auto-oxidation, and at most it reached 70% of the rate measured after addition of the supercomplex.

### Flash Photolysis and Flow-flash Experiments

The purified samples (typical protein concentrations were in the range of 2–3 μM and 1–1.5 μM for *bc*_1_-Cyt*c*O and CytcO, respectively) were transferred into a Thunberg cuvette and the atmosphere was exchanged for N_2_ on the vacuum line. The sample was reduced by the addition of 1 μM phenazine methosulfate and 5 mM sodium ascorbate from the sidearm of the cuvette. In order to achieve complete reduction of all the redox-active centers in samples containing supercomplexes, 0.5–1 mM sodium dithionite (Merck Millipore) was added and the reduction state was confirmed spectrophotometrically. After complete reduction, the atmosphere was exchanged for CO on a vacuum line. To measure the pH dependence of the reaction of the reduced Cyt*c*O or cyt. *bc*_1_-Cyt*c*O with O_2_, the sample was prepared in 10 mM Tris-HCl, the atmosphere was replaced consecutively by N_2_ and then CO, and the sample was reduced by 4 mM ascorbate, 1 μM PMS and 100 μM dithionite at pH 7.5. Samples at different, higher pH values were prepared by adding various volumes of 1 M Tris-HCl buffer at pH 10 followed by incubation for at least 1 hour. Alternatively, the sample in 10 mM Tris-HCl at pH 7.5, was reduced with 4 mM ascorbate, 1 μM PMS and 100 μM dithionite (under CO atmosphere) and then mixed with an O_2_-saturated solution at different pH values containing 100 mM buffer. The buffering capacity of the O_2_ solution was much higher (100 mM) than that of the enzyme solution (10 mM) so the final pH after mixing was determined by the former. The pH after mixing was measured using a pH meter.

The CO rebinding/recombination kinetics to the catalytic site was measured as a change in absorbance over time at several wavelengths after the photolysis by a ~10-ns laser flash (λ = 532 nm, Nd-YAG laser, Quantel; the flash-photolysis/flow-flash setup was purchased from Applied Photophysics UK). The time resolution of the set-up was ~10^−7 ^s. The absorbance changes were then fitted to an exponential decay function using the ProK software from Applied Photophysics U.K.

In order to determine the CO concentration dependence of the CO-recombination, 1 ml of a protein sample was transferred into a Thunberg cuvette and treated as described above, except that after reduction it was left under a nitrogen atmosphere. The sample was covered with a layer of paraffin oil and CO was added in small aliquots of CO-saturated buffer (100 mM Tris-HCl (pH 7.5), 100 mM NaCl, 0.015% (w/v) DDM) with a gas-tight Hamilton syringe through the paraffin layer. The CO-recombination kinetics was studied after each CO addition using a flash-photolysis set-up (Applied Photophysics, UK).

In flow-flash experiments, the reduced and CO-blocked protein sample was mixed 1:3 with oxygen-saturated buffer (~1.2 mM O_2_) in a flow-flash setup (Applied Photophysics, UK.). About 200 ms after mixing (mixing time <10 ms) with the oxygenated buffer, CO was dissociated from the catalytic site by a short laser pulse (~10-ns laser flash (λ = 532 nm, Nd YAG-laser, Quantel). Changes in absorbance were recorded over time at different wavelengths. The data were fitted to a kinetic model using the ProK software from Applied Photophysics, UK.

## Additional Information

**How to cite this article**: Graf, S. *et al*. Rapid Electron Transfer within the III-IV Supercomplex in *Corynebacterium glutamicum. Sci. Rep.* **6**, 34098; doi: 10.1038/srep34098 (2016).

## Supplementary Material

Supplementary Information

## Figures and Tables

**Figure 2:**
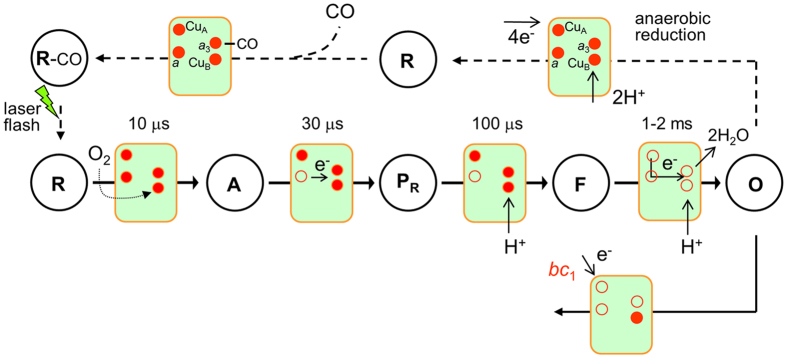
Schematic illustration of the reactions in Cyt*c*O studied in this work. The four red circles represent the redox-active metal sites as indicated (filled - reduced). During preparation of the sample, the oxidized Cyt*c*O (**O**) is reduced by 4 electrons under an atmosphere of pure N_2_ (anaerobic reduction) to yield the fully reduced Cyt*c*O (state **R**). The sample is then incubated under an atmosphere of CO, which binds to heme *a*_3_ at the catalytic site forming **R**-CO (see dashed line). The reaction studied in this work (indicated by a solid line) is started by photo-dissociation of the CO ligand (laser flash), yielding the reduced Cyt*c*O (**R**), which allows O_2_ to bind to heme *a*_3_ forming state **A** with a time constant of ~10 μs at 1 mM O_2_. An electron is then transferred from heme *a* to the catalytic site forming the “peroxy” state called **P**_**R**_ with a time constant of ~30 μs. Next, a proton is taken up from solution forming the ferryl state **F** with a time constant of ~100 μs. In most oxidases studied to date, at the same time the electron at Cu_A_ equilibrates with heme *a*. This electron transfer is not indicated in the figure because in the *C. glutamicum* Cyt*c*O the equilibrium is shifted towards reduced Cu_A_. In the final step of the reaction the electron from Cu_A_ (or the Cu_A_-heme *a* equilibrium) is transferred to the catalytic site forming the oxidized Cyt*c*O (state **O**) in 1–2 ms. In cyt. *bc*_1_-Cyt*c*O supercomplex, as soon as Cu_A_ is partly oxidized (τ ≅ 100 μs), electrons are transferred from the cyt. *bc*_1_ complex to the Cyt*c*O. Further electron transfer from the *c* and *b* hemes occurs over a slower time scale. For simplicity, all these electron transfers are indicated schematically in the lower part of this scheme (the time constants are given in Fig. 8). The pumped protons are not shown.
