# Supplementary material for: Rapid Electron Transfer within the III-IV Supercomplex in Corynebacterium glutamicum
Source: Sci Rep. 2016 Sep 29;6:34098. doi: 10.1038/srep34098 (PMC5040959; doi:10.1038/srep34098)
Supplement: Supplementary Information [file srep34098-s1.doc]

Supplementary Material

Rapid Electron Transfer within the III-IV Supercomplex in *Corynebacterium glutamicum*

Simone Graf^1,2^, Olga Fedotovskaya^1^, Wei-Chun Kao^3^, Carola Hunte^3^, Pia Ädelroth^1^, Michael Bott^4^, Christoph von Ballmoos^2^ and Peter Brzezinski^1^*

^1^ Department of Biochemistry and Biophysics, The Arrhenius Laboratories for Natural Sciences, Stockholm University, SE-106 91 Stockholm, Sweden.

^2^ Department of Chemistry and Biochemistry, University of Bern, Freiestrasse 3, 3012 Bern, Switzerland

^3^ Institute of Biochemistry and Molecular Biology, ZBMZ, Faculty of Medicine, BIOSS Centre for Biological Signalling Studies, University of Freiburg, 79104 Freiburg, Germany

^4^ IBG-1: Biotechnology, Institute of Bio- and Geosciences, Forschungszentrum Jülich, Wilhelm-Johnen-Strasse, D-52425 Jülich, Germany


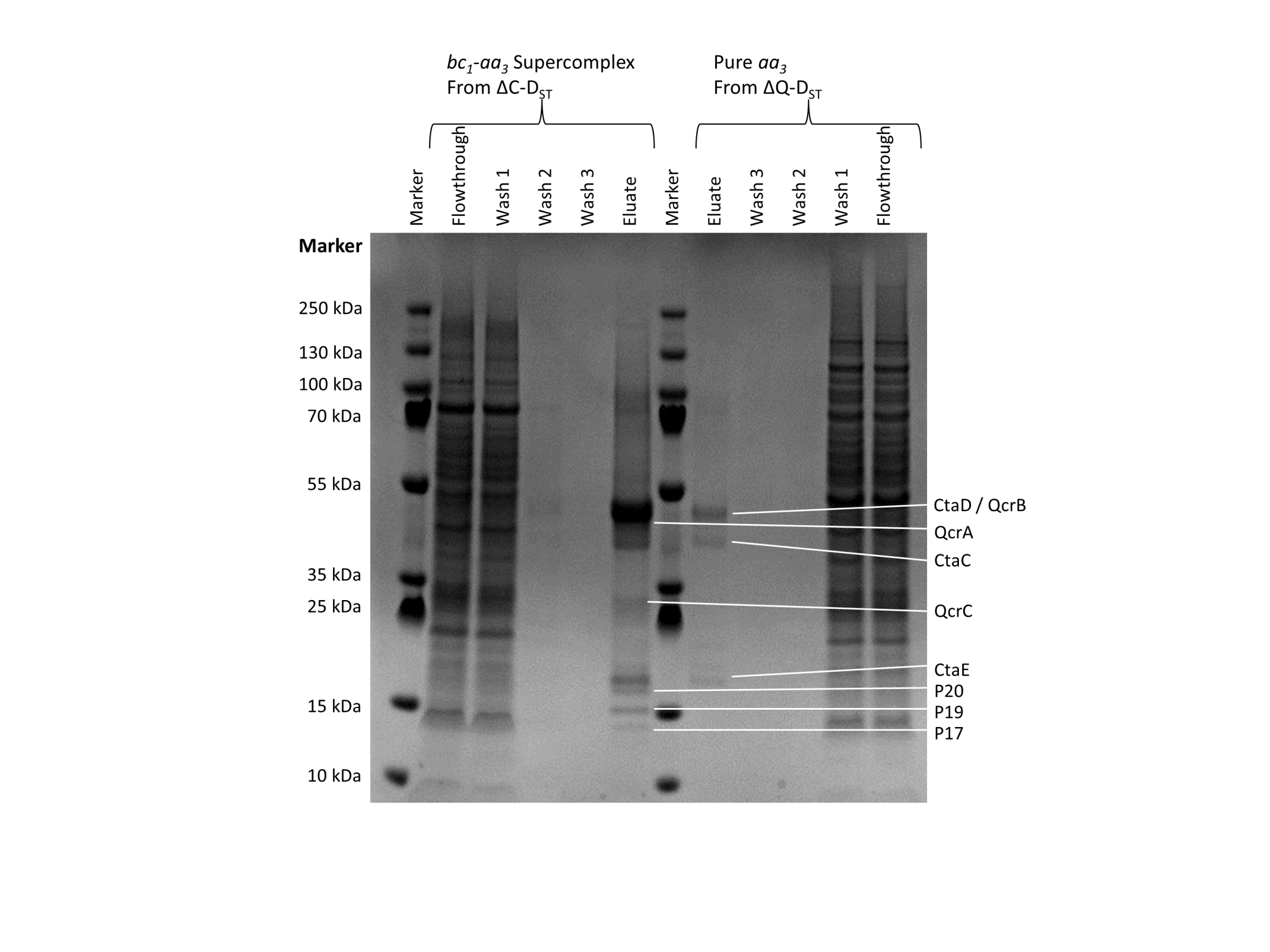


Figure S1. SDS PAGE of the purifications via Gravity flow Strep-Tactin Superflow column from membranes originating from the *C. glutamicum* strains ΔC-D_St_ and ΔQ-D_St_, respectively. The eluate of the purification from the ΔC-D_St_ strain displayed all the subunits for the cyt. *bcc* complex, the three core subunits of the cytochrome c oxidase, as well as some additional proteins of unknown function (P20,P19, and P17), previously discussed and identified in (Niebisch & Bott (2003) *J. Biol. Chem*. 278, 4339). The eluate for the purification from ΔQ-D_St_ membranes contained only the three core subunits of the Cyt*c*O.

Figure S2. Dithionite-reduced minus ferricyanide-oxidized difference spectra of the purified Cyt*c*O (black) and the purified *bc*_1_-Cyt*c*O (red). The samples were oxidized with a few microliters of 1 mM ferricyanide and incubated at room temperature for 15 minutes, until the absorbance was stable. A spectrum of the oxidized Cyt*c*O was recorded. Then, the sample was reduced by sodium dithionite after which a spectrum of the reduced Cyt*c*O was recorded.

**Figure S3.** The reduced CO-bound minus reduced difference spectra for the purified cyt. *bc*_1_*-*Cyt*c*O supercomplex (red), and purified Cyt*c*O (black). The CO concentration was ~1 mM.

Figure S4. Quinol oxidase activity of purified *bc*_1_*-*Cyt*c*O supercomplex measured at different time points after purification when the sample was stored at 4⁰C between measurements. The activity was measured using a Clark-type O_2_-electrode. The activity is given as electrons transferred per second per molecule of Cyt*c*O. Background oxygen consumption due to auto-oxidation of the quinol was subtracted. The background O_2_-reduction rate in the presence of quinol was 5-10 % of that measured after addition of the enzyme (this background "activity" was subtracted).

**Figure S5**. CO-concentration dependence of the CO-recombination rate constant for the purified Cyt*c*O (**A**) and the *bc*_1_-Cyt*c*O supercomplex (**B**). The second-order rate constants were 7.6±0.2 10^3^ M^‑1^s^‑1^ and 9.4±0.2 10^3^ M^‑1^s^‑1^ for the Cyt*c*O and cyt. *bc*_1_*-*Cyt*c*O complex, respectively.

Figure S6. Reaction of the purified *bc*_1_-Cyt*c*O supercomplex with O_2_ at different KCl concentrations. Changes in the absorbance are shown at 445 nm. All the traces are normalized to the initial CO-dissociation absorbance change at *t*=0. Conditions: 100 mM Tris-HCl at pH 7.5 and KCl as indicated.

Figure S7. Reaction of the purified *bc*_1_*-*Cyt*c*O supercomplex with O_2_ monitored at 445 nm for different NaCl concentrations. Conditions: 100 mM Tris-HCl at pH 7.5, 100 mM KCl and NaCl as indicated. The inset shows the time constant of the first visible kinetic transition (A --> P_R_) as a function of NaCl concentration. The other components displayed Na^+^-independent rates.
